# Level of Stigma among Spanish Nursing Students toward Mental Illness and Associated Factors: A Mixed-Methods Study

**DOI:** 10.3390/ijerph16234870

**Published:** 2019-12-03

**Authors:** Julián Rodríguez-Almagro, Antonio Hernández-Martínez, David Rodríguez-Almagro, José Miguel Quiros-García, María del Carmen Solano-Ruiz, Juan Gómez-Salgado

**Affiliations:** 1 Department of Nursing, Ciudad Real Nursing Faculty, University of Castilla-La Mancha, 13071 Ciudad Real, Spain; antomatron@gmail.com; 2 Department of Emergency, Hospital General of Ciudad Real, 13005 Ciudad Real, Spain.; josemiguelquiros@hotmail.com (J.M.Q.-G.); 3 Department of Nursing, Alicante Nursing Faculty, University of Alicante, 03080 Alicante, Spain; carmen.solano@ua.es; 4 Department of Sociology, Social Work and Public Health, University of Huelva, 21071 Huelva, Spain; salgado@uhu.es; 5 Safety and Health Postgraduate Programme, Universidad Espíritu Santo, Guayaquil 091650, Ecuador

**Keywords:** nursing students, nurses, mental illness, mental health, public health, stigma, mixed methods

## Abstract

Mental health problems have been identified by the World Health Organization as a global development priority. Negative attitudes toward mental health patients have been documented in multiple health professionals. The aim of this study was to determine the level of stigma and associated factors toward people with mental health problems among students doing their degree in nursing. An explanatory sequential mixed-methods approach. A cross-sectional descriptive observational study was carried out on a sample of 359 students doing their degree in nursing. Students had to be enrolled in any of the four years of study of the degree at the time the questionnaire was done. We explored the perception and experience of students doing their degree in nursing regarding the level of stigma, through in-depth interviews (*n* = 30). The mean overall Mental Health Stigma Scale (MHSS) score was 30.7 points (SD = 4.52); 29.5% (*n* = 106) scored low for stigma, 49.9% (*n* = 179) showed moderate stigma, and 20.6% (*n* = 74) scored high. The multivariate analysis showed that 4th-year students had an OR of 0.41 (CI95%: 0.20–0.84) for high/moderate stigma and that 3rd-year students had an OR of 0.49 for high/moderate stigma compared with 1st-year students. We also observed that students with family members with mental health problems had an OR of 2.05 (CI95%: 1.19–3.56) for high/moderate stigma compared with students who did not have family members with mental health problems. The following categories emerged: fear and lack of knowledge, breaking the silence, and integration into society. The levels of mental health stigma in our sample of nursing students were moderate. Stigma levels were lower in 3rd- and 4th-year students (i.e., after having received training in mental health), and in students with family members with mental health problems.

## 1. Introduction

Mental health problems have been identified by the World Health Organization as a global development priority [1]. However, despite progress in medical specialties and health sciences when it comes to treating different conditions, the stigma associated with mental health problems is a factor that continues to increase [2] It is important to assess the impact of dealing with stigma, both for caregivers and those with mental illness [3,4,5,6,7,8,9,10,11]. The World Health Organization defines stigma as a ‘mark of shame, disgrace, or disapproval’ [12]. The negative consequences of stigmatising attitudes include ‘being rejected, discriminated against and excluded from participating in a number of different areas of society’. Furthermore, being stigmatised not only affects the psychological well-being and development of people with mental disorders, but also acts as a significant barrier to seeking, accessing and adhering to treatment [13].

It has been noted that mental health professionals can have negative attitudes toward both mental illness and the patients themselves [14,15]. Manifestations of these stigmatising attitudes by health professionals can be a barrier to the provision of appropriate services and care for this population [16]. The Spanish Neuropsychiatry Association (AEN), in its 2008 *Consenso sobre promoción de salud, prevención del trastorno mental y disminución del estigma* (Consensus on health promotion, mental health prevention and stigma reduction) [17], stated that stigma is very common. It could thus become a significant obstacle to rehabilitating and integrating people with mental health problems in Spain [2,18,19]. Furthermore, negative perceptions of people with mental health problems leads to stigmatising and discriminatory attitudes and behaviour toward them [20].

Other research [21] confirms that stigma affects these people’s social relationships and the perception they have of themselves. In fact, serious mental illnesses and people who suffer from them are viewed with a certain amount of prejudice by the general public and there is a real lack of knowledge, both in terms of symptoms and their progression and the different treatment options [22]. The existence of stigma toward mental illnesses has also been documented in the university population, more specifically in studies related to health sciences [23,24,25]. Different methods to fight stigmatising attitudes in the general population, in students, and in health professionals have been described [26]. The main strategies are education and contact with people with experience in mental illnesses [27].

Some researchers have recommended that stigma be discussed among health sciences students so that they can go help create awareness and act to help society overcome stigma and discrimination [28]. The stigma of mental illness also has implications for nursing, especially for nursing students [29]. Nursing students provide nursing care to persons with mental illnesses, and their attitudes towards them become the main determinants of the quality and outcomes of care these patients receive [30]. Harbouring negative views towards people would influence the way nurses view their patients and the nature of their work itself. This could not only affect their role as an advocate in reducing stigma, but also hinder the development of therapeutic relationships with patients [31,32]. Therefore, nursing education and placements play a major role in shaping the attitudes held by nursing students towards people with mental disorders. By providing enough and in-depth exposure to theoretical and practical knowledge, a more positive attitude towards mental health nursing could be developed [33]. Furthermore, there is currently limited evidence on the stigma and recovery attitudes of nursing students before and after a traditional mental health clinical placement. To improve future undergraduate nursing education, it is vital to understand the impact that existing education and clinical placement has on undergraduate students’ attitudes to mental health consumers and mental health nursing [34].

The objective of our study was to determine the level of stigma and associated factors toward people with mental health problems among students doing their degree in nursing. For this purpose, we used a mixed-methods study to understand these factors among nursing students in both qualitative and quantitative ways.

## 2. Materials and Methods

### 2.1. Design and Selection of Study Subjects

The study design is mixed methods, with a quantitative part and a qualitative part, with the aim of integrating theoretical perspectives and improving understanding.

Mixed-methods research is increasingly being used in the sciences to gain a more complete understanding of clinical issues and hear the voices of participants [35,36]. Mixed-methods research involves the collection and analysis of both qualitative and quantitative data and their integration. Drawing on the strengths of both approaches [37,38] is a way to facilitate the integration of qualitative and quantitative data in mixed-methods studies [39].

On the one hand, we use a quantitative methodology with a deductive-type approach with the aim of testing our hypothesis, gathering descriptive information, and examining the relationship between variables, giving us measurable evidence.

On the other hand, and based on the results of the quantitative methodology, we will extract a series of questions with a qualitative methodology using an inductive-type approach based on the context and on the significance of human experience, or phenomenology. In our case, these questions took the form of in-depth interviews that helped us to obtain detailed information about the participants. 

### 2.2. Methodology of the Quantitative Phase

#### 2.2.1. Study Design and Scope

An observational, descriptive, cross-sectional study on a non-probabilistic sample of 359 students doing their degree in nursing at the Faculty of Nursing of Ciudad Real (Spain) was conducted between December 2017 and February 2018. 

To take part in the study, students had to be enrolled in any of the four years of study of the degree at the time the questionnaire was done. Only questionnaires that had no missing data were included in this analysis. Only five students left items unanswered and were excluded.

The first contact with the students was made through an e-mail informing them of the objective of the work and the anonymous and voluntary nature of their participation; all those who responded that they wanted to participate were sent a link to an online questionnaire. Students completed the questionnaire in their free time.

#### 2.2.2. Information Sources

A data collection booklet was designed that included an explanation of the objectives and purpose of the study and ensured anonymity, confidentiality and other ethical guarantees. This questionnaire was sent to those responsible for each year of study at the faculty in question, who then forwarded them to the students to be completed on a voluntary basis. 

The anonymous questionnaire gathered demographic information on the sample: age, gender, year of study, rotations in mental health, existence of family members with mental health problems, and existence of friends with mental health problems.

To assess mental health stigma, a validated Spanish translation of the anonymous Mental Health Stigma Scale (MHSS) Appendix A was used, composed of 12 Likert-type questions, which students scored on a Likert scale, from 1 (strongly disagree) to 5 (strongly agree) [40]. The MHSS tool was validated, obtaining Cronbach’s alpha values between 0.67 and 0.74, with a positive correlation with another mental health and stigma scale (*r* = 0.58, *p* < 0.01) [41].

The scores ranged from 12 to 60 points. Scores below 28 were considered low stigma and scores above 35 were considered high stigma.

#### 2.2.3. Calculation of the Sample Size

The following criteria were used to calculate the sample size: Prevalence of high stigma: 15% [40], confidence level of 95%, precision (absolute error) of 3% and a population of 425 enrolled students. Based on these criteria, a minimum of 239 students was needed.

#### 2.2.4. Statistical Analysis Used

For the descriptive statistics, absolute and relative frequencies were used for the qualitative variables. Means (standard deviation) were used for the quantitative variables if they had a normal distribution; medians (interquartile range) were used if they did not. Then, multivariate analysis was performed using binary logistic regression to control bias and confounding, where the dependent variable was the overall score of the dichotomised stigma index (moderate‒high stigma ≥29 points/low stigma <29 points). The independent variables were age, gender, year of study, having done a clinical rotation in mental health, having family members with mental health problems, and having friends with mental health problems. The program SPSS v. 24.0 was used for the statistical analysis. 

#### 2.2.5. Ethical Approval

This observational study, done using anonymous data, was designed in accordance with the Declaration of Helsinki developed by the World Medical Association. The study was approved by the research committee of the University (UCLM) with code number C2-2017. Besides the in-person explanation, the questionnaire included a summary of the study purpose and informed consent.

### 2.3. Methodology of the Qualitative Phase

A study with a qualitative methodology was done using in-depth interviews with nursing students. Qualitative methodology is especially useful for understanding a phenomenon from the point of view of those involved, by exploring their beliefs, expectations and feelings and explaining the reasons behind their behaviours and attitudes. 

This was a convenience sampling [42], interviewing students who contacted the research team after having seen appeals made by the faculty between December 2017 and January 2018; although the size of the theoretical sample was not sufficient to guarantee external validity in terms of other research models [43], it was sufficient to saturate all categories, including participants with diverse sociodemographic characteristics. The interviews were carried out in Spanish.

The data were gathered through interviews, which were recorded in password-protected audio files that only the authors had access to [44].

The only criteria for inclusion were being a nursing degree student aged between 18 and 75 years.

A total of 30 in-depth interviews were carried out and a series of sociodemographic characteristics were collected, including age and gender, following a semi-structured script that we created based on the quantitative phase in order to modulate the interviews. The interviews lasted 30 to 45 min and were transcribed in full. All participants took part voluntarily and in an informed manner. The anonymity of participants and the confidentiality of the information were maintained. The students were identified with codes to ensure their anonymity, identifying each interview with the letter I (for Interview) followed by a sequential number from 1 to 30. 

All interviews began with an open-ended question [45], ‘what does working/living/knowing people with a mental health problem mean to you?’ to invite participants to narrate their experiences with people with a mental health problem and in order to focus the research phenomenon. The participants were encouraged to freely talk about their experiences, and the interviewer followed the script freely to encourage the participants. The interviews were carried out in a classroom at the Nursing School of Ciudad Real.

A descriptive, qualitative, phenomenological design was used based on the Giorgi method. The aim of this approach is to describe the meanings of the phenomenon from the perspective of the subjects’ life experience [45], through key themes. This method provided a description of the experience of students making initial contact with people with a mental health problem by categorising all findings into units of sensation, based on the philosophy of Edmund Husserl and Merleau-Ponty, and is generic enough to be applied to any science [46].

The Atlas.ti programme (Scientific Software Development GmbH, Berlin, Germany) was used for the qualitative analysis. During the process, the criteria of methodological rigour and credibility, auditability and transferability were taken into account [47].

## 3. Results

### 3.1. Results of the Quantitative Phase

Ultimately, 84.5% (*n* = 359) of enrolled students correctly completed the questionnaire, with a mean age of 20.0 years (interquartile range = three years); 83.0% (*n* = 298) of participants were female, 11.1% (*n* = 40) had done a clinical rotation in mental health, 30.4% (*n* = 77) stated that they had a family member with mental health problems and 21.4% (*n* = 109) stated that they had friends with mental health problems. The most important characteristics are shown in Table 1.

#### 3.1.1. Students’ Level of Stigma toward Patients with Mental Health Problems

**Figure 1:**
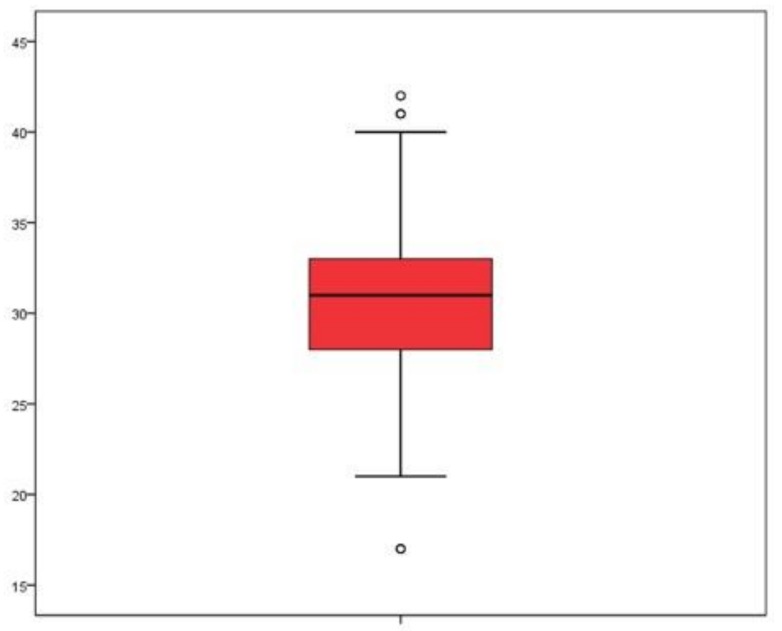
Boxplot of students’ level of stigma toward patients with mental health problems.

**Figure 2:**
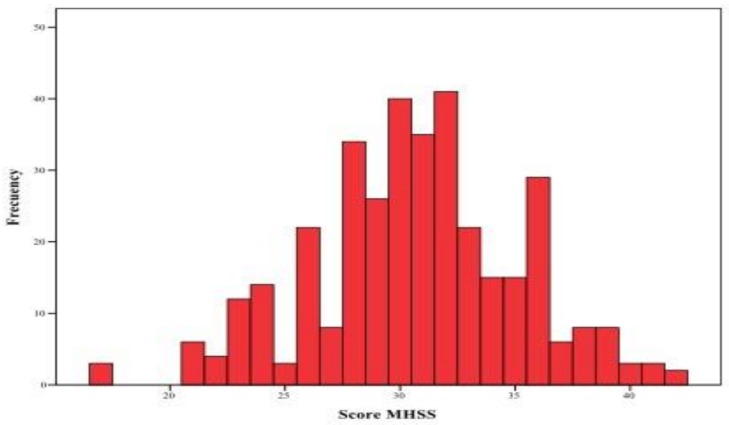
Histogram of students’ level of stigma toward patients with mental health problems.

The mean overall MHSS score was 30.7 points (SD = 4.52).;29.5% (*n* = 106) scored low for stigma, 49.9% (*n* = 179) had moderate stigma scores, and 20.6% (*n* = 74) scored high for stigma (Figure 1 and Figure 2).

#### 3.1.2. Factors Associated with Stigma toward Mental Health Problems and Mental Health Patients

The multivariate analysis using binary logistic regression showed that 4th-year students had an OR of 0.41 (CI95%: 0.20–0.84) for high/moderate stigma and that 3rd-year students had an OR of 0.49 for high/moderate stigma compared with 1st-year students. No differences were observed between 2nd-year and 1st-year students. (Table 2)

We also observed that students with family members with mental health problems had an OR of 2.05 (CI95%: 1.19–3.56) for high/moderate stigma compared with students who did not have family members with mental health problems (Table 2).

No correlation was observed with gender, having friends with mental health problems, or having done clinical rotations in mental health (Table 2).

### 3.2. Results of the Qualitative Phase

The objective of the qualitative phase was to add results with greater depth and obtain more detailed understanding of students’ experiences of rotations and their approaches to patients with a mental health problem, aiming to get them to explain their life experience with mental illnesses in their own words. The interview uses open-ended questions that are partly derived from the results obtained in the quantitative phase of the MHSS questionnaire. All interviews began with the question ‘what does working/living/knowing people with a mental health problem mean to you?’, which served to get the students talking about their experiences.

#### 3.2.1. Participants

A total of 30 students were interviewed, with a mean age of 20.2 years, very similar to the mean age of the quantitative sample, which was 20 years. After rigorously examining the interviews, three categories emerged representative of the experience of being a nursing degree student and of their relationship with patients with a mental health problem. These categories were named as follows: fear and lack of knowledge, breaking the silence, and integration into society. 

#### 3.2.2. Fear and Lack of Knowledge

The interviews with the students show that there is fear and a lack of knowledge toward mental health problems in the general population and in hospitalised patients in particular.

“For me, stigma in mental health is a sort of imaginary mark on the person suffering from mental illness, which society sees, senses and rejects. It is a clear form of discrimination based on the population’s fear and lack of knowledge of these issues.” (I2)

They tell us that there is a certain amount of rejection toward people with mental health problems and talk about the reasons behind this rejection and discrimination, mentioning that self-stigma may even occur.

“Stigma in mental health is rejection or isolation of people with a mental illness. It is due to prejudices, stereotypes or a lack of understanding that people have about people with mental illnesses, classifying them as violent, incompetent, irresponsible or unable to carry out their day-to-day activities.” (I5)

“Stigma is a label we give to the mentally ill which first and foremost denotes inferiority with regard to the person with the label and toward the society they live in. What’s more, it is often the mentally ill themselves that give themselves these labels.” (I19)

#### 3.2.3. Breaking the Silence

From the interviews, the opinion emerged that society should seek to support people with a mental health problem and show that it is no different from any other type of health problem:

“It is also key that we break the silence surrounding mental illnesses, which are currently in the shadows, and which are illnesses like any other. Mental health is very important, and anyone can experience mental health problems.” (I23)

In their interviews, they told us how we can try to break this stigma by appropriating the media and using it in our favour, as it is often the media that labels people and distorts reality:

“One aspect that I think is important in the stigmatisation of mental health is the influence of the media, which often broadcasts images that distort and aggravate the problems these people have, giving a false image of what it means to be mentally ill.” (I15)

“Scientific evidence, on the other hand, tells us that people with serious mental illnesses are no more violent than other people. Instead, they tend to be victims and not attackers. Their illness makes them into a subject of disdain, mockery and violence, something which does not occur with other illnesses.” (I9)

Another contrast we see in the analysis of the interviews is that eradication is the responsibility of society as a whole, as anyone can suffer from mental illness:

“Anyone can suffer from mental illness; eradicating stigma is our collective responsibility.” (I28)

#### 3.2.4. Integration into Society

One expectation that emerges in almost all of the interviews is in relation to the integration of people with mental health problems into society, at all levels, whether personally or professionally:

“We need to change the way people think and show them that they are just like anybody else, because nobody wants to be treated like that.” (I6)

Students call for more social education through existing media: 

“as health professionals, we need to eliminate all stigma and help raise awareness among the population, increase knowledge, and eliminate misinformation through health education in the form of campaigns, talks and so on.” (I11)

Encouraging behaviour that promotes social integration must be pursued while guaranteeing rights and, above all, dignity:

“The main objective is to end stigmatisation and encourage the integration of people with mental illnesses, guaranteeing their rights and dignity.” (I21)

Another notable piece of data is that there is constant reference to the fact that people do not choose to get an illness, of any type. We should therefore treat people as we would like to be treated, as anyone can suffer from illness of any kind:

“People with a mental illness have not chosen their illness, they are people like anyone else, with dreams, fears, needs and so on, who we should give opportunities to so they can live a life that is as normal as possible.” (I7)

“Treat people as you’d like to be treated.” (I14)

According to our results in the qualitative phase, we can say that the image of people with mental illnesses in society continues to be skewed toward them being dangerous, which is reinforced by the images of mental illnesses we see in the news. We must start by changing the way in which these types of illnesses are talked about in the media, i.e., from a neutral point of view, which would lead to better integration of mentally ill people into society.

## 4. Discussion

In this study, we evaluated, in a global manner, the level of stigma toward people with mental health problems among students doing their degree in nursing and identified the factors determining or associated with stigma. Our methodological approach, combining a qualitative process and a quantitative process, allowed us to collect the real experiences of students [35,36]. The analysis of our results shows that manifestations of stigma were lower in students in a higher year of study, coinciding with previous studies [34,48,49,50]. However, there is existing literature that states the opposite, highlighting that health professionals are not immune to stigmatising the patients they work with [14,51]. 

We therefore agree that it is important to consider the type of teaching that professionals receive when it comes to designing actions to reduce stigma. In the case of nursing, we can observe that stigma reduces as students advance in their degree due to the training students receive in their 3rd year, which is when they study a module in psychiatry and mental health, as happens in previous studies [34,52]. Thus, we could say that more theoretical training and longer clinical placements are associated with more positive student attitudes towards mental health nursing, as happens in previous studies [50,53].

Another key finding was that manifestations of stigma were lower in students with a family member with a mental health problem. In our data, this fact acts as a protective factor, as it did in prior studies [34,48]. In line with Barrett and Jackson [54], we can say that early reduction of stigma within the nursing syllabus could be key to increasing nursing students’ confidence while at the same time encouraging changes in stigmatising attitudes, as occurs in our results. We think it is important for students who have not had any prior experience and who do not know anyone with a mental illness to engage with these types of illnesses. This is supported by our qualitative results, in which people with a mental illness are depicted as being dangerous; this needs to change, by changing the image of people with mental illnesses in society.

Previous studies [33,34] show that qualitative research on what can be learnt from students’ personal experiences with family and friends with mental illnesses is needed. This approach will influence students’ attitudes and help students who have not experienced this situation. Our results show that students’ personal experiences with a family member or friend with mental illness affect their relationships with people with mental health problems, thus covering the gap that Foster et al. [25] highlighted in their study.

Our qualitative results indicate that the change in stigmatising attitudes toward people with mental illnesses would be lower in students with a positive attitude toward people with a mental health problem, as occurs in the work of Madigan [55]. Our qualitative results are in line with those from previous studies [48,49] when dealing with an existing fear and lack of knowledge towards mental health problems in the general population and in hospitalised patients in particular.

One of the limitations of the present study is the small sample size. We believe that future studies should include a higher number of students, as it is important to be able to compare with students from other faculties and from other disciplines within the health sciences. The findings may not apply in other contexts and settings. Another limitation of the study is social desirability: one might think that since the students were studying nursing, they may have a sense that they should not stigmatize people with mental illness. Therefore, they may try to hide their true feelings about stigmatizing people with mental illness.

What is true, however, is that this study shows us the strong points of mixed methods in health sciences. The mixed-methods strategy gave a broad overview of the experience of being a nursing degree student and of their relationship with patients with mental health problems. Another strength is having carried out the study using mixed methods, approaching the research problem in two distinct but complementary ways, as the quantitative and qualitative data facilitated the interpretation of the experiences of our participants. 

### Relevance for Clinical Practice

Our findings point to the need for the design and development of an awareness programme on mental illnesses aimed at these students. It is important that students present a low level of stigma towards this type of patient in order to be provide them with the best possible quality of care.

Direct contact through an intervention with mental patients and nursing students would be effective in the short term to improve the levels of stigma towards mental patients, and could easily be included in training programmes targeted at nursing students.

## 5. Conclusions

Levels of mental health stigma in our sample of nursing students were moderate and paradoxically were linearly lower at higher years of study and in students with family members with mental health problems. With regard to the analysis of the interviews, we can say that there is greater stigma due perhaps to a lack of knowledge of these types of illnesses in society, although all interviewees agree that an increase in education through the media could improve and even eradicate this stigma, and that we need to demonstrate that nobody chooses to have an illness, so we should treat others as we would like to be treated, as anyone can suffer from mental illness. It is necessary to determine nursing students’ beliefs and attitudes toward people with mental health problems with the objective of helping them develop skills that facilitate the professional care of these patients. Actions or programmes to reduce stigma toward mental health problems and mental health patients should be implemented at educational centres, especially those where health professionals are trained

## Figures and Tables

**Table 1:** Characteristics of the study subjects and relationship with stigma level.

Independent Variables	*N* = 359 (*n*/%)	Low Stigma (*n* = 106) (<29 Points) (*n*/%)	Moderate‒high Stigma (*n* = 253) (≥29 points) (*n*/%)
**Gender**			
Male	61 (17.0)	18 (29.5)	43 (70.5)
Female	298 (83.0)	88 (29.5)	210 (70.5)
Year of study			
First year	91 (25.3)	19 (20.9)	72 (79.1)
Second year	96 (26.7)	29 (30.2)	67 (69.8)
Third year	84 (23.4)	28 (33.3)	56 (66.7)
Fourth year	88 (24.5)	30 (34.1)	58 (65.9)
Clinical rotation in mental health			
No	319 (88.9)	94 (29.5)	225 (70.5)
Yes	40 (11.1)	12 (30.0)	28 (70.0)
Having family members with mental health problems			
No	250 (69.6)	83 (33.2)	167 (66.8)
Yes	109 (30.4)	23 (21.1)	86 (78.9)
Having friends with mental health problems			
No	282 (78.6)	87 (30.9)	195 (69.1)
Yes	77 (21.4)	19 (27.7)	758 (75.3)

**Table 2:** Multivariate analysis of factors associated with stigma in mental health.

	Logistic Regression Probability of High/Moderate Stigma	
Variables	*P*-Value	OR CI95%
Gender	0.518	
Male (Reference cat.)		1
Female		1.23 (0.65–2.33)
Year of study		
First year (Reference cat.)		1
Second year	0.151	0.61 (0.31–1.20)
Third year	**0.043**	**0.49 (0.24–0.98)**
Fourth year	**0.015**	**0.41 (0.20–0.84)**
Clinical rotation in mental health	0.995	
No (Reference cat.)		1
Yes		1.00 (0.46–2.18)
Having family members with mental health problems	**0.010**	
No (Reference cat.)		1
Yes		**2.05 (1.19‒3.56)**
Having friends with mental health problems	0.300	
No (Reference cat.)		1
Yes		1.37 (0.76–2.48)

## Appendix A. Mental Health Stigma Scale (MHSS)

1. Someone who is recovering from a mental health problem can look after their children alone.
2. Someone who is recovering from a mental health problem should remain in hospital or in supported housing.
3. Someone who is recovering from a mental health problem can live alone.
4. People with mental health problems have the right to adopt children.
5. Discipline/education at home can prevent the majority of mental health problems in children.
6. People with a lack of drive tend to have mental health problems.
7. Someone with a lack of discipline/education is more likely to have a mental health problem.
8. People with mental health problems are more likely to be violent.
9. I would be worried if a mental health hospital was opened near to where I live.
10. It would be difficult for me to establish a friendship with someone with a mental health problem.
11. For me, being admitted to a mental health hospital would be a sign of having failed in life.
12. It would be difficult for me if a friend found out that one of my family members had a mental health problem.
